# Hydro-organic mobile phase and factorial design application to attain green HPLC method for simultaneous assay of paracetamol and dantrolene sodium in combined capsules

**DOI:** 10.1186/s13065-023-00990-7

**Published:** 2023-08-02

**Authors:** Nora A. Abdallah, Manar M. Tolba, Amina M. El-Brashy, Fawzia A. Ibrahim, Mona E. Fathy

**Affiliations:** Department of Pharmaceutical Analytical Chemistry, Faculty of Pharmacy, Mansoura University, Mansoura, 35516 Egypt

**Keywords:** Experimental factorial design, Eco-friendly analysis, Paracetamol, Dantrolene sodium, HPLC, Greenness assessment tools

## Abstract

The greenness of any analytical method has become a very important aspect of a good analytical method. However, most chromatographic methods depend on the usage of relatively large amounts of lethal and un-decaying chemicals and solvents. So, a green approach based on the full factorial design was employed to develop a simple and rapid HPLC technique for concurrent determination of paracetamol and dantrolene sodium in their combined capsules. Both drugs are highly recommended to be administered together in patients with severe musculoskeletal disorders. Avoiding the routine methodology and resorting to the modern technology represented in the usage of experimental design allows rapid determination of the studied drugs using the optimum quantity of chemicals to avoid any waste of resources. Simultaneous separation of a binary mixture of paracetamol and dantrolene sodium was accomplished using a reversed phase Hypersil C18 column using an eco-friendly isocratic eluent. The used mobile phase consisted simply of ethanol: water (40:60, v/v). Orthophosphoric acid was used to adjust the pH of the mobile phase to 4.5. Triethanolamine (0.2%) was added aiming to reduce the peak tailing. The assay was completed within less than 6 min adopting 0.8 mL/min as a flow rate. The detection was carried out using a UV-detector at 290 nm. The suggested technique shows a linear correlation over concentration ranges of 1.0–200 and 1.0–40 µg/mL for paracetamol and dantrolene sodium, respectively. The suggested technique allowed the simultaneous analysis of the two co-formulated drugs in their synthetic mixture and combined capsule. The suggested technique is considered a greener substitute for the other reported HPLC techniques through the usage of safer solvents and chemicals, along with decreasing both waste output and analysis time. The method is accurate with recoveries between 97.85 and 101.27%, precise, as %RSD for the intraday and interday precision were between 0.39 and 1.72% and very sensitive with limits of detection (LOD)’s 0.15 and 0.18 µg/ml and limits of quantification (LOQ)’s 0.48 and 0.61 µg/ml for paracetamol and dantrolene sodium, respectively. The method greenness was ensured through its assessment by four greenness metrics. It is also validated following the International Conference on Harmonization Guidelines. The recommended technique could be a good alternative to traditional methods in the routine quality control analysis of the studied drugs due to its minimum harm to the planet or human beings.

**Supplementary Information:**

The online version contains supplementary material available at 10.1186/s13065-023-00990-7.

## Introduction

**Fig. 1:**
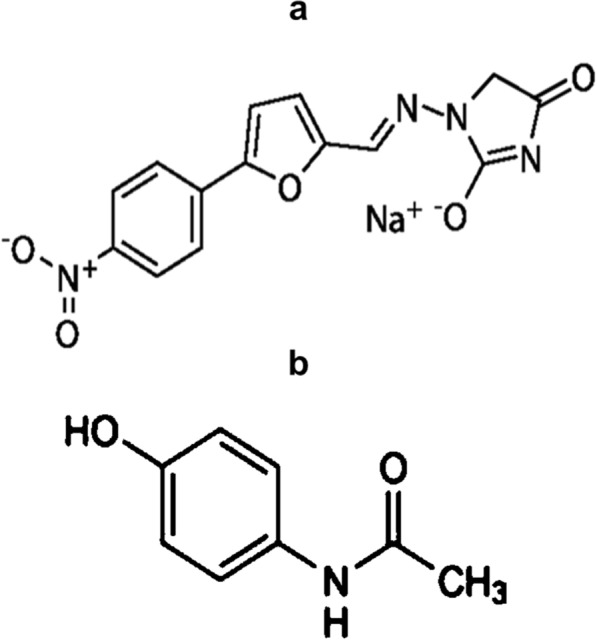
The Chemical structural of **a** dantrolene sodium **b** paracetamol

Paracetamol and dantrolene sodium are used in combination for the treatment of musculoskeletal disorders as their combined form is superior to a single agent alone [1]. Both drugs should be considered a wise therapeutic option for patients with acute pain in the lower back [2]. Dantrolene Sodium (DAN); (Fig. 1a) is the hemi heptahydrate of the sodium salt of 1-[5-(4-nitrophenyl) furfurylideneamino] imidazolidine-2, 4-dione. DAN is a muscle relaxant drug which acts on skeletal muscles. It separates muscular contraction from excitation by interrupting the calcium release from the sarcoplasmic reticulum [3]. Paracetamol (PAR); (Fig. 1b) is 4´-Hydroxyacetanilide; N-(4-Hydroxyphenyl) acetamide. It has analgesic, antipyretic effects and some anti-inflammatory properties [3].

Green analytical chemistry (GAC) started to attract attention in 2000s [4, 5]. This developing field is associated with optimizing the standards of analytical procedures through the decreased usage of hazardous solvents and increased safety for analysts and the planet earth [6, 7]. It has recently been preferred not only for pharmaceutical analyses but also for food analyses [8, 9]. Food analysis and quality control can be safely done by employing greener alternative techniques instead of traditional analytical methods, which require tedious, time-consuming sample preparation and are frequently linked to environmental pollution [10]. The success of any technique in science and technology is measured by its simplicity, environmentally friendly, and its applications [8]. HPLC is considered one of the most frequently used techniques in pharmaceutical field, especially in the analysis of drugs in pharmaceutical preparations. However, HPLC methods usually consume large quantities of organic hazardous solvents that may have a damaging influence on the environment and the analyst [11]. Most mobile phases contain methanol and acetonitrile as organic solvents. Although these solvents have astonishing elution abilities, there are some concerns related to their negative effect on humanity safety. The suggested technique employs the hydro-organic mobile phase which is composed of water and ethanol as a greener substitute for the unsafe traditional mobile phases.

Experimental design (DOE) is a process that depends mainly on making systematic plans that make full use of minimum experimentation to obtain maximum information. A full factorial design (FFD) is a type of DOE ‘multivariate optimization’ which allows investigating the effect of all the factors simultaneously based on the responses of the dependent factors and the interactions between the independent factors [12]. FFD ensures optimal performance and reliability of the used parameters and the results of the proposed method [13].

The aim of this research is to present a modern chromatographic method by introducing a greener and non-toxic mobile phase as a substitute for the traditionally quite unsafe ones. This can be achieved via the usage of ethanol [14], while maintaining the method performance unaffected. Ethanol is a good greener substitute to methanol and acetonitrile [15] as declared by American Chemical Society Green Chemistry Institute. Ethanol is the most trusted solvent from the viewpoint of environmental standard solvent guide [16, 17]. Also, the usage of the experimental design allows the usage of optimum chemicals which decreases the waste and enhances the method greenness [18]. Herein, a validated, rapid, green and sensitive HPLC technique is presented for simultaneous determination of PAR and DAN in their combined capsules. The determination of the binary mixture was done by employing an eluent that consisted of water: ethanol (60:40, v/v, pH 4.5, adjusted by phosphoric acid and 0.2% triethanolamine (TEA)). The separation of the binary mixture was accomplished in a very short time, less than 6 min. Literature review revealed that the separation of that mixture was performed by other researchers using different chromatographic methods, such as TLC densitometry and HPLC [19, 20], spectrophotometric methods [20–24]. Most of the optimized separation methods cited in the literature for the analysis of the studied drugs by RP-HPLC involve studying of a large number of variables in the separation process. In addition, those methods use large quantities of organic solvent in the mobile phase, which produces a negative effect on the environment. For this reason, it is needed to design a more effective, green and time-saving method using the experimental design procedure.

To the best of our knowledge, no research involving a full factorial design experiment for the separation of these drugs has been reported. This was motivation to look for a greener solvent, such as ethanol, to separate that binary mixture. The proposed technique was found to be less time-consuming and more eco-friendly when compared to others. The greenness metric reports were used as a reference to compare the suggested method with those earlier reported HPLC methods [19, 20].

## Experimental

### Instruments and software

Knauer Chromatograph equipped with a Knauer, D-14163 injector valve with a 20 µL loop (Berlin, Germany) was used. Eluent was filtered using 0.45 µm membrane filters (Millipore, Cork, Ireland). Consort NV P-901 calibrated pH–Meter (Belgium) was used for pH measurements. Sonication was done by Digital Ultrasonic Cleaner, Model: Soner 206 H, MTI Corporation (USA). Factorial design statistical analysis was done using Minitab^®^ 16.2.0 software, USA.

### Materials and solvents

Authentic samples of PAR and DAN were provided from Alexandria, Eva-Pharma Co., and Chemipharm Pharmaceutical Industries, Cairo, Egypt, respectively. HPLC grade ethanol was bought from Fischer Scientific (USA). Triethanolamine (≥ 99.5%) was bought from Sigma Aldrich (Germany). Orthophosphoric acid (85%, w/v) was obtained from Riedel-deHäen, Honeywell Research Chemicals (Germany). Dantrelax compound^®^ capsules, batch no. # 201123A containing 25 mg DAN and 300 mg paracetamol/capsule, are product of Chemipharm Pharmaceutical Industries and were purchased from a local Egyptian pharmacy.

### Standard solutions

200.0 μg/mL of both PAR and DAN were prepared separately in the mobile phase as stock solutions. Working standard solutions were prepared on demand by further dilution of different volumes of the stocks with mobile phase. The prepared stock solutions were stored at 4 °C in the fridge and remained valid for 2 weeks.

### General procedures

#### Construction of calibration graphs

Accurately measured volumes of both PAR and DAN standard solutions were moved into separate two sets of 10 mL volumetric flasks. The flasks were completed to the mark with mobile phase to obtain the concentration range of the two drugs: (1.0–200.0 and 1.0–40.0 µg/mL for PAR and DAN, respectively). 20 µL of the previously prepared solutions were introduced into the sample loop, injected into the column and eluted under the formerly adjusted parameters. Finally, the calibration graphs were performed by plotting the area under the peak Vs concentration of the drugs in µg/mL and the regression equations for each drug were derived.

#### Assay of the PAR and DAN in the Synthetic mixtures

Synthetic mixtures of PAR and DAN with a ratio of 12:1, respectively, which is the ratio in their co-formulated capsule, were prepared. These solutions were then treated as mentioned under “2.5.1. Construction of the calibration graphs”. The percentages found of PAR and DAN were then calculated referring to the calibration graphs or the regression equations.

#### Assay of the binary mixture in their co-formulated capsules

The content of ten capsules of Dantrelax Compound^®^ were carefully weighed and thoroughly mixed. An accurately weighed amount of the powder equivalent to one capsule was moved into 100.0 mL measuring flask and about 40.0 mL of ethanol were added. The flask was subjected to sonication for thirty minutes to ensure thorough mixing of the contents. Then, the flask was completed to full volume with water. Finally, the mentioned procedure under “2.5.1. Construction of calibration graphs” was performed. The capsule contents of the two drugs were calculated referring to the calibration graphs or regression equations.

#### Experimental design

**Fig. 2:**
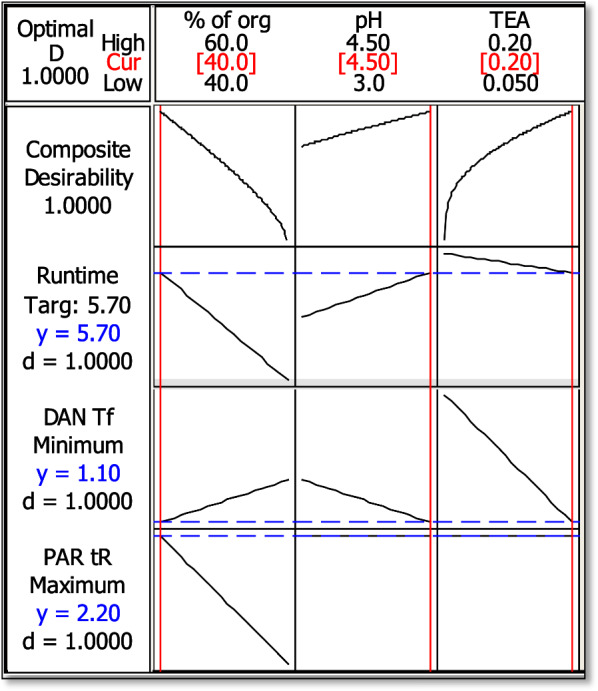
2^3^ full factorial design (FFD) optimization plot

Experimental design is a process that depends mainly on making systematic plans that make full use of minimum experimentation to obtain maximum information then employing it using statistical models to make significant conclusions from the obtained results [25]. Multilevel factorial design, 2^3^ FFD was applied in this study for determination of the optimal conditions that produced the ideal response values. Minitab optimizer is provided with upper, target, and lower values for each response (retention time of PAR, tailing factor of DAN peak and retention time of DAN). Minitab calculates the optimum requirements of organic solvent, % of TEA and pH and draws a plot. The optimization plot displays the influence of each factor (column) on the responses (rows), as shown in Fig. 2. The optimization plot shows the effect of each parameter on the responses and chooses the optimum of each factor for best responses. All details of how to carry out DOE process and how it calculated the optimum conditions are explained in detail in EL-Shorbagy et al. [26].

## Results and discussion

The proposed method presents a green, fast, sensitive and economic RP-HPLC technique for resolving a binary mixture used for treatment of muscle spasms related diseases like lower back pain. The proposed technique employs factorial design to optimize and maintain the optimum parameters used for separation; hence it saves time and resources.

### Method development and optimization

**Fig. 3:**
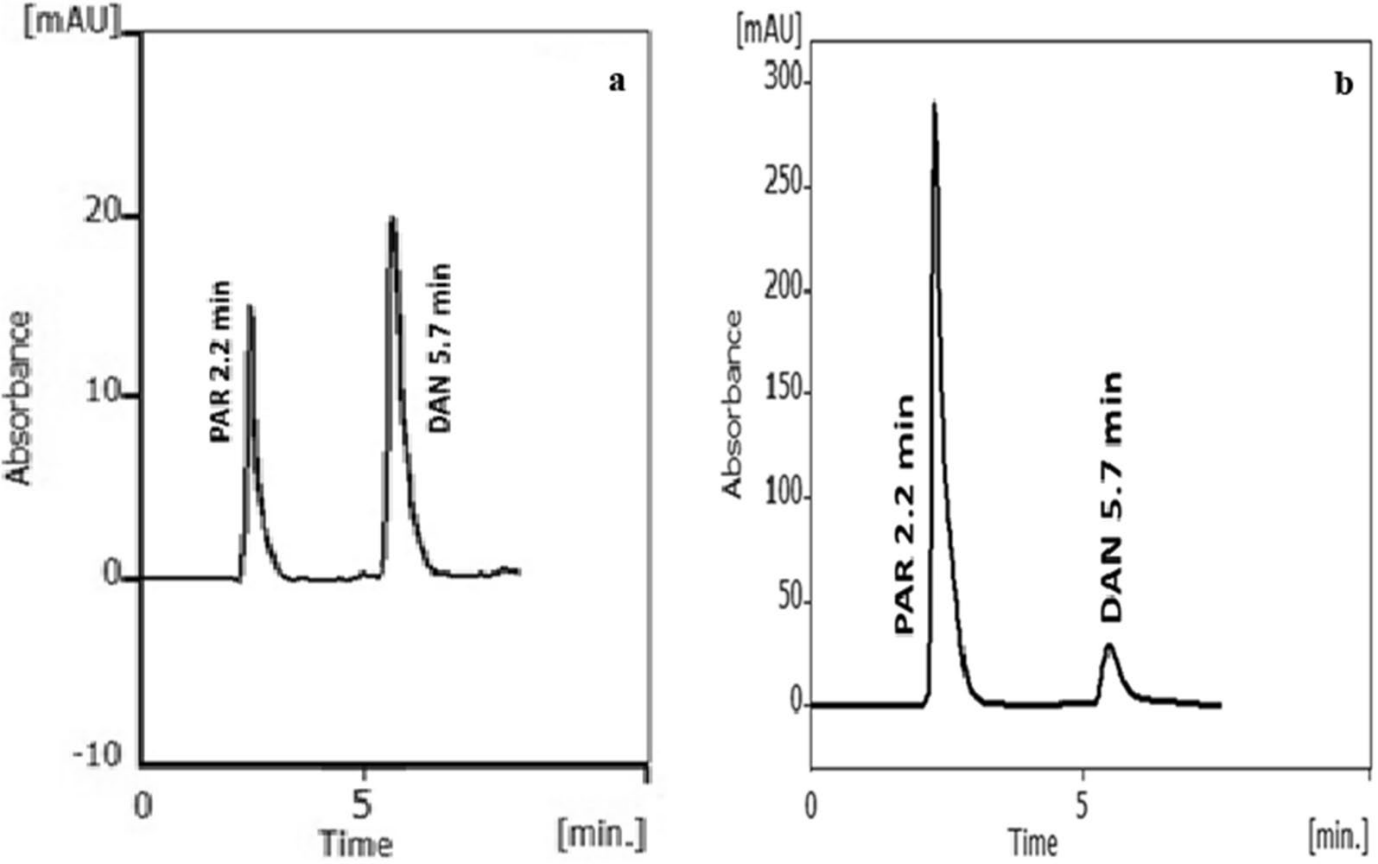
**a** A typical chromatogram of synthetic mixture of PAR and DAN in 1:1 ratio (30.0 µg/mL each) under the described chromatographic conditions. **b** A Chromatogram of the PAR (200.0 µg/mL) and DAN (16.66 µg/mL) in their co-formulated capsule under the described chromatographic conditions

Different parameters were investigated for the sake of obtaining the optimized ratios of mobile phase and suitable column that produces good separation without wasting any extra solvents or chemicals using 2^3^ FFD to ensure the reliability and optimal performance of the method. Good optimization led to decreasing the environmental hazards through the usage of eco-friendly and relatively safe solvents, such as ethanol and water. The optimization also resulted in shrinking the required time for chromatographic analysis and consequently reducing waste production while maintaining the best resolution and sensitivity. Typical chromatogram of symmetrical peaks of a synthetic mixture of PAR and DAN is shown in Fig. 3a. The chromatographic parameters adopting the optimum conditions were calculated and shown in Table 1. The two drugs were well resolved and separated using isocratic elution of an aqueous mobile phase consisting of 40% ethanol, 60% water and 0.2% TEA, in less than 6 min.

**Table 1:** Chromatographic conditions and Parameters of system suitability of the proposed HPLC method for the determination of PAR and DAN

Chromatographic conditions	Column	Hypersil C18 column	
	Mobile phase	40:60 (v/v) of ethanol: water, 0.2% TEA at pH 4.5 $\pm$ 0.02 and 0.8 mL/min flowrate	
	Detection	UV detection 290 nm	
Parameters of system suitability			
Parameter		PAR	DAN
No of theoretical plates, N		1936	4500
Selectivity factor, α		12.66	
Resolution, R_s_		8.75	
Retention time (t_R_), min		2.2	5.7
Tailing factor (T), 5% of the peak height		0.97	1.14
Asymmetry factor, 10% of the peak height		1.11	1.21
Run time, min		6	

#### Selection of suitable column

Three columns were put on trial for choosing the best one for separation of PAR and DAN including:

1. Hypersil BDS Cyano LC Column (250 × 4.6 mm, 5 μm).
2. Hypersil Phenyl LC Column (250 × 4.6 mm, 5 μm).
3. Thermo scientific Hypersil C18 column (150 mm × 4.6 mm i.d., 5-µm).

The third column (Hypersil C18 column) was found to be the best one regarding the resolution of the peaks and run time.

#### Selection of suitable wavelength

**Fig. 4:**
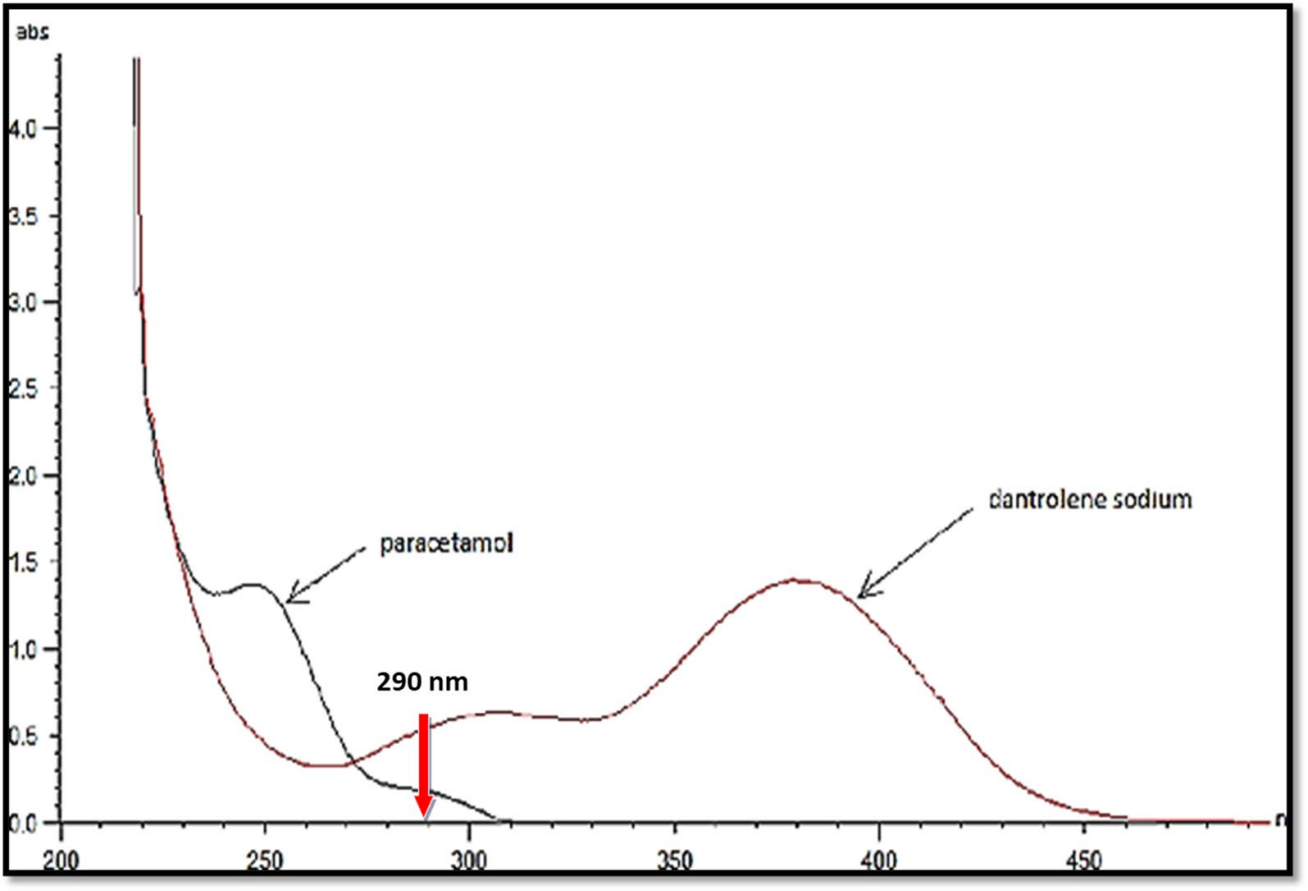
Zero order absorption spectra of DAN (20 μg/mL) and PAR (14 μg/mL)

UV detection was carried out at 290 nm. This choice was adopted based on the UV spectra of PAR and DAN [21] as shown in Fig. 4. The spectra showed that 290 nm is the most suitable especially for DAN, as it has the lowest amount in the capsule and thus higher sensitivity is required.

#### Eluent composition (screening experiment)

This method was mostly directed at avoiding the use of hazardous solvents and using ethanol as a green organic solvent for RP-HPLC. Ethanol is believed to be a safe and less hazardous eluent, as it is distinguished by its high viscosity, low vapor pressure consequently a less evaporation and inhalation potential, thus reducing the necessity of thorough waste cleaning. All these advantages give superiority to ethanol for usage in mobile phase [27]. Also, the usage of hydro-organic mobile phases as ethanol/water mixtures allows decreasing the amount of organic solvent essential to achieve separation [28]. Compared to acetonitrile and methanol, ethanol has lower disposal costs. This is mostly because of its environmentally compatible waste, especially with the high expenses associated with the other solvents waste disposal [29].

The ratios of ethanol and triethanolamine (TEA) in the eluant and its pH were studied to choose the best separation of the studied drugs in the shortest possible time using experimental design.

Different ethanol percentages (10–60%) were tested and the results of the studied dependent parameters were inserted to Minitab. Addition of triethanolamine was very important for DAN elution and its peak shape, as the mobile phases missing TEA led to tailing in DAN peak. Thus, various concentrations of TEA from 0.05 to 0.3% were tried individually. Lesser amount of TEA caused insufficient improvement of DAN peak shape, while increasing its concentration caused shorter retention time, which negatively affects the resolution of the peaks. Different pH values (3.0–6.0) were also tested to study their effect on the separation. The pH of the mobile phase had no effect on the peak shape or retention time of PAR. However, DAN peak became closer to PAR with pH values less than 4.0. Meanwhile, pH greater than 5 increases the DAN retention time.

#### Experimental design

The main goal of experimental designs was to reach the optimum conditions with the minimum number of trials needed while examining the maximum number of factors. Some initial chromatographic experiments were required before performing an experimental design to determine the chromatographic factors which have significant effect on the chromatographic responses (screening experiment). In this experiment, three factors were found to affect the chromatographic performance including: % of organic modifier, ethanol, TEA and pH. These factors were mainly affecting the retention time of DAN, the retention time of PAR and DAN tailing factor, as shown in Table 2. From the previous experiments, 2^3^ full factorial design was applied for optimization of the current study using two level combinations and three independent factors (pH and % of ethanol and TEA). FFDs are the form of factorial designs in which all influencing independent factors (k) with (m) level combinations are investigated. The number of experimental runs needed for a FFD depends on the number of independent factors (k) to be studied. As a general rule, the design requires a total of m^k^ experiments [30] to be performed. From the screening step, it was found that the optimum input ranges in the 2^3^ FFD design is as follows: organic modifier in the range of 10–60%, the % of TEA was between 0.1 and 0.25% and a pH in the range of 4.0–5.0. These critical factors were inserted into Minitab software to find their optimum conditions. The design suggested a set of 8 experiments (Additional file 1: Table S1) are needed to represent interactions of the mentioned factors and their effects on selected chromatographic responses (t_R_ of DAN, t_R_ of PAR and T_f_ of DAN). For the choice of the most critical factors influencing the method, a synthetic mixture containing 30 µg/mL of each drug was prepared. The suggested eight runs were carried out, then the obtained chromatograms were interpreted and the results were inserted into Minitab software to determine the dependent factors. Finally, response optimizer compromise between different responses then the optimum setting of the input variables and hence desirability values were determined. In response optimizer; lower, target and upper values are defined for dependent responses. Optimal setting for the input variables along with desirability values are calculated by Minitab response optimizer. To ensure that the optimum conditions are obtained, Minitab response optimizer calculates the composite desirability (D) which evaluates if the responses are in their acceptable limits and it ranges from zero to one. Zero is not accepted as it means that many of the responses are out of their accepted limits, while one means that the condition reached is optimum, so its value is better to be one or near one (Table 2). According to the response optimizer and optimization plot (Fig. 2), it was proven that the optimal chromatographic conditions were 40.0% v/v for ethanol, 0.20%v/v TEA and pH of 4.5. Pareto charts in Additional file 1: Fig. S1 showed the effect of the factors on the responses. It was found that % of the organic modifier highly affected the retention time of PAR. Additional file 1: Figs. S2 and S3 illustrate the interaction plots and the main effect plots of the independent factors on the dependent ones.

**Table 2:** Response optimization of 2^3^ full factorial design for HPLC–UV separation of PAR and DAN

Factor	Goal	Lower	Target	Upper	Weight	Import	Predicted responses	Desirability
t_r_ PAR	Maximize	2.1	2.2	2.2	1	1	2.2	1.000
t_f_ DAN	Minimize	1.1	1.1	1.4	1	1	1.1	1.000
t_r_ DAN	Target	4.0	5.7	7.2	1	1	5.7	1.000
Optimum Conditions: 40% ethanol, 0.2% TEA and pH 4.5								Composite Desirability (D) = 1.000

Finally, the used mobile phase consisted of 40:60 (v/v) of ethanol: water, 0.2% TEA at pH 4.5  ± 0.02 and 0.8 mL/min flowrate at 290 nm UV detection were employed to allow simultaneous analysis of the two drugs with acceptable sensitivity. The adopted chromatographic conditions are summarized in Table 1.

### Suggested technique validity

The proposed technique was validated according to International Conference on Harmonization Guidelines (ICH) [31].

#### Linearity, limit of quantitation (LOQ) and limit of detection (LOD)

The linear range of quantification for PAR and DAN was studied adopting the proposed method and the results were presented in Table 3. Statistical analysis of the produced data [32], proved the linearity of the calibration graphs. Linear regression equations of PAR and DAN were as follow:

$$\begin{aligned} {\rm AUP}\,=\,22.784\,+\,7.058\, {\rm C} \,& \quad ({\rm r}\,=\,0.9999) \,{\rm for\, PAR} \\ {\rm AUP}\,=\,19.476\,+\,24.975\,{\rm C}\,& \quad ({\rm r}\,=\,0.9999)\, {\rm for\, DAN }\end{aligned}$$

Where: AUP is the area under the peak, C is the concentration in µg/mL and r is the correlation coefficient.

**Table 3:** Analytical performance data for the determination of the PAR and DAN by the proposed HPLC method

Parameter	PAR	DAN
Linearity range (µg/mL)	1.0–200	1.0–40.0
Intercept (a)	22.784	19.476
Slope (b)	7.058	24.975
Correlation coefficient (r)	0.9999	0.9999
S.D. of residuals (Sy/x)	1.99	5.446
S.D. of intercept (Sa)	0.806	3.068
S.D. of slope (Sb)	0.010	0.147
Percentage relative standard deviation, % RSD	1.133	1.28
Percentage relative error, % Error	0.356	0.48
Limit of detection, LOD (µg/mL)	0.15	0.18
Limit of quantitation, LOQ (µg/mL)	0.48	0.61

The limits of detection and quantitation were calculated practically following signal to noise ratio as in USP [33] and the results are shown in Table 3.

#### Accuracy

Statistical analysis was applied for comparison between the obtained results from the suggested method and those by the official USP methods [33] adopting the Student *t* test and the variance ratio *F* test [32]. The official reference methods adopted HPLC technique to assay each of PAR and DAN. The results showed that there was not a significant difference between the performance of both methods in terms of accuracy and precision, respectively (Table 4).

**Table 4:** Assay results for the determination of PAR and DAN in pure form by the suggested and official USP methods

Compound	Suggested technique			Official USP method [33]
	Concentration taken (µg/mL)	Concentration found (µg/mL)	% Found	% Found
PAR	1.0	0.98	98.00	100.57
	2.0	1.957	97.85	99.11
	5.0	4.911	98.22	100.37
	10.0	9.957	99.57	
	20.0	20.066	100.33	
	30.0	29.804	99.35	
	40.0	40.506	101.27	
	60.0	59.823	99.71	
	100.0	100.388	100.39	
	200.0	199.782	99.89	
Mean			99.46	100.02
± S.D			1.13	0.792
*t* test			0.79 (2.20) *	
*F* test			2.03 (19.38) *	
DAN	1.0	0.981	98.10	100.76
	2.0	2.016	100.80	98.88
	5.0	5.026	100.52	100.45
	10.0	9.806	98.06	
	20.0	20.116	100.58	
	30.0	30.322	101.07	
	40.0	39.745	99.36	
Mean			99.78	100.03
± S.D			1.28	1.01
*t* test			0.29 (2.31) *	
*F* test			1.62 (19.33) *	

*N.B.* Each result is the average of three separate determinations

^*^The figures between parentheses are the tabulated *t* and *F* values at *P* = 0.05 [32]

#### Precision

Two levels precision were performed on each drug by examining them on three successive times in the same day or on three successive days to test intra-day and inter-day precision, respectively and the precision results are shown in Table 5.

**Table 5:** Precision data for the determination of PAR and DAN by the suggested HPLC technique

Drug	Conc. (μg/mL)	Intra-day			Inter-day		
		Mean ± S.D	%RSD	% Error	Mean ± S.D	%RSD	% Error
PAR	20.0	100.59 ± 0.70	0.71	0.40	99.33 ± 1.01	1.02	0.59
	60.0	99.88 ± 0.39	0.39	0.23	100.12 ± 1.72	1.72	0.91
	100.0	100.12 ± 0.62	0.63	0.36	100.39 ± 0.92	0.92	0.53
DAN	8.0	100.34 ± 0.91	0.91	0.52	99.66 ± 1.06	1.06	0.61
	12.0	98.72 ± 0.78	0.78	0.46	100.43 ± 1.12	1.12	0.64
	20.0	99.83 ± 0.75	0.76	0.44	99.59 ± 0.89	0.89	0.52

*N. B.* Each result is the average of three separate determinations

#### Robustness

Some chromatographic conditions were subjected to minor changes to test the proposed method robustness. Those changes were carried out univariately. The investigated variables were pH of the mobile phase (4.5 ± 0.1), ethanol percentage (40 ± 1%) and TEA concentration (0.2 ± 0.01%) as shown in Table 6. The proposed method was proven to be robust as such minor changes did not affect either the resolution or the area under peak of the two drugs.

**Table 6:** Robustness of the suggested technique using 20.0 µg/mL of PAR and DAN

Parameter	Concentration found (µg/mL)		% Found	
	PAR	DAN	PAR	DAN
*Ethanol ratio, %*				
39	20.091	19.946	100.46	99.73
40	19.845	20.045	99.23	100.23
41	19.724	19.968	98.62	99.84
Mean			99.44	99.93
± S.D			0.94	0.26
%RSD			0.94	0.26
%Error			0.54	0.15
*pH*				
4.4	19.768	19.874	98.84	99.37
4.5	20.056	19.963	100.28	99.81
4.6	20.137	20.011	100.69	100.06
Mean			100.49	99.75
± S.D			0.29	0.35
%RSD			0.29	0.35
%Error			0.20	0.20
*TEA, %*				
0.19	19.921	20.056	99.61	100.28
0.2	20.079	20.017	100.41	100.08
0.21	19.840	20.084	99.20	100.42
Mean			99.74	100.26
± S.D			0.62	0.17
%RSD			0.62	0.17
%Error			0.36	0.11

*N.B.* Each result is the average of three separate determinations

#### System suitability

System suitability assessments were done referring to the USP [33] and ICH Guidelines [31] on mixture of PAR and DAN to calculate the chromatographic parameters. The obtained parameters are presented in Table 1.

### Applications

The proposed technique was employed effectively to analyze both PAR and DAN simultaneously in their synthetic mixture and combined capsule as shown in Tables 7 and 8, respectively. The results in both were in a great agreement with those obtained adopting the official USP methods [33] in regards to accuracy and precision [32]. Chromatogram for the two studied drugs in their combined capsule is illustrated in Fig. 3b.

**Table 7:** Assay results for the determination of PAR and DAN in synthetic mixtures of their pharmaceutical ratio (12:1) by the suggested HPLC technique

PAR/DAN ratio	Suggested technique						Official USP method [33]	
	Concentration taken (µg/mL)		Concentration found (µg/mL)		% Found		% Found	
	PAR	DAN	PAR	DAN	PAR	DAN	PAR	DAN
12:1	60.0	5.0	61.008	4.999	101.68	99.98	99.68	101.14
	120.0	10.0	120.084	9.889	100.07	98.89	100.68	100.69
	180.0	15.0	177.966	15.207	98.87	101.38	99.37	98.58
	192.0	16.0	193.018	15.920	100.53	99.50	101.05	99.43
Mean				100.29	99.94	100.20	99.96	
± S.D				1.16	1.06	0.69	0.91	
*t* test				0.13	0.03		(2.45)*	
*F* test				2.12	1.21		(9.28)*	

*N. B.* Each result is the average of three separate determinations

^*^The figures between parentheses are the tabulated *t* and F values at *P* = 0.05 [32]

**Table 8:** Assay results for the determination of PAR and DAN in their combined capsules by the suggested HPLC technique

PAR/DAN ratio	Suggested technique						Official USP method	
	Concentration taken (µg/mL)		Concentration found (µg/mL)		% Found		% Found	
	PAR	DAN	PAR	DAN	PAR	DAN	PAR	DAN
Dantrelax compound® 300PAR/25DAN	120	10	117.648	9.865	98.04	98.65	98.04	100.75
	150	12.5	152.325	12.606	101.94	100.85	101.94	98.69
	200	16.67	199.659	16.489	99.83	99.96	99.83	101.37
Mean					99.94	99.82	100.01	100.27
± S.D					1.95	1.11	0.66	1.15
*t* test					0.06	0.44	(2.78)*	
*F* test					5.84	1.60	(19.00)*	

*N. B.* Each result is the average of three separate determinations

^*^The figures between parentheses are the tabulated *t* and* F* values at P = 0.05 [32]

### Greenness estimation

Although the studies focused on eliminating the waste and adopting ecofriendly and sustainable methods [17, 34, 35] were started in 1995, they were not assessed by the analytical society. One of the priorities of green analysis is to reduce the use of harmful substances without affecting the efficiency of the chromatographic performance [36]. The usage of environmentally friendly solvents in the mobile phase is one of the most important ways to obtain greener analysis [37]. The goal of this work is to declare that traditional quite dangerous techniques can be replaced by ecofriendly ones while maintaining same analytical behavior.

Recently, green analysis as well as indexing the method greenness has become very important. Indexing the method greenness allows the possibility of ranking the methods according their greenness which is very helpful [16, 38, 39]. Four assessing methods were employed to assess the greenness of the recommended technique and compare it with reported ones.

First, National Environmental Methods Index (NEMI) has been applied on the proposed and reported methods. NEMI is a tool using greenness profile and regarded as one of the first appeared methods [16]. Table 9 shows that the proposed method achieves the four criteria of the greenness profile and is greener than the reported HPLC methods according to NEMI profile. Water and ethanol are neither classified as PBT nor hazardous by the EPA’s Toxic Release Inventory [17, 34], the pH of the mobile phase is not corrosive and the waste is less than 50 g/run.

**Table 9:** Comparison of greenness report between the suggested and reported HPLC methods adopting NEMI method [16]

Mixture	Method	Mobile phase	Run time (min.)	Flow rate (mL/min.)	Waste (g/run) Run time × flow rate [10]	Greenness profile**
Paracetamol and Dantrolene	Reported HPLC method [19]	Methanol: water (55:45, v/v) pH 3 with aqueous formic acid	12	1.0	12	
Paracetamol and Dantrolene	Reported HPLC method [20]	Methanol: potassium dihydrogen phosphate (50:50 v/v) pH 3.5 by phosphoric acid	9	1.0	9	
Paracetamol and Dantrolene	Proposed HPLC method	Ethanol: water (40:60) pH 4.5 by phosphoric acid	6	0.8	4.8	

^**^Four key terms are referred to PBT (persistent, bio-accumulative, and toxic), Hazardous, Corrosive, and Waste

**Fig. 5:**
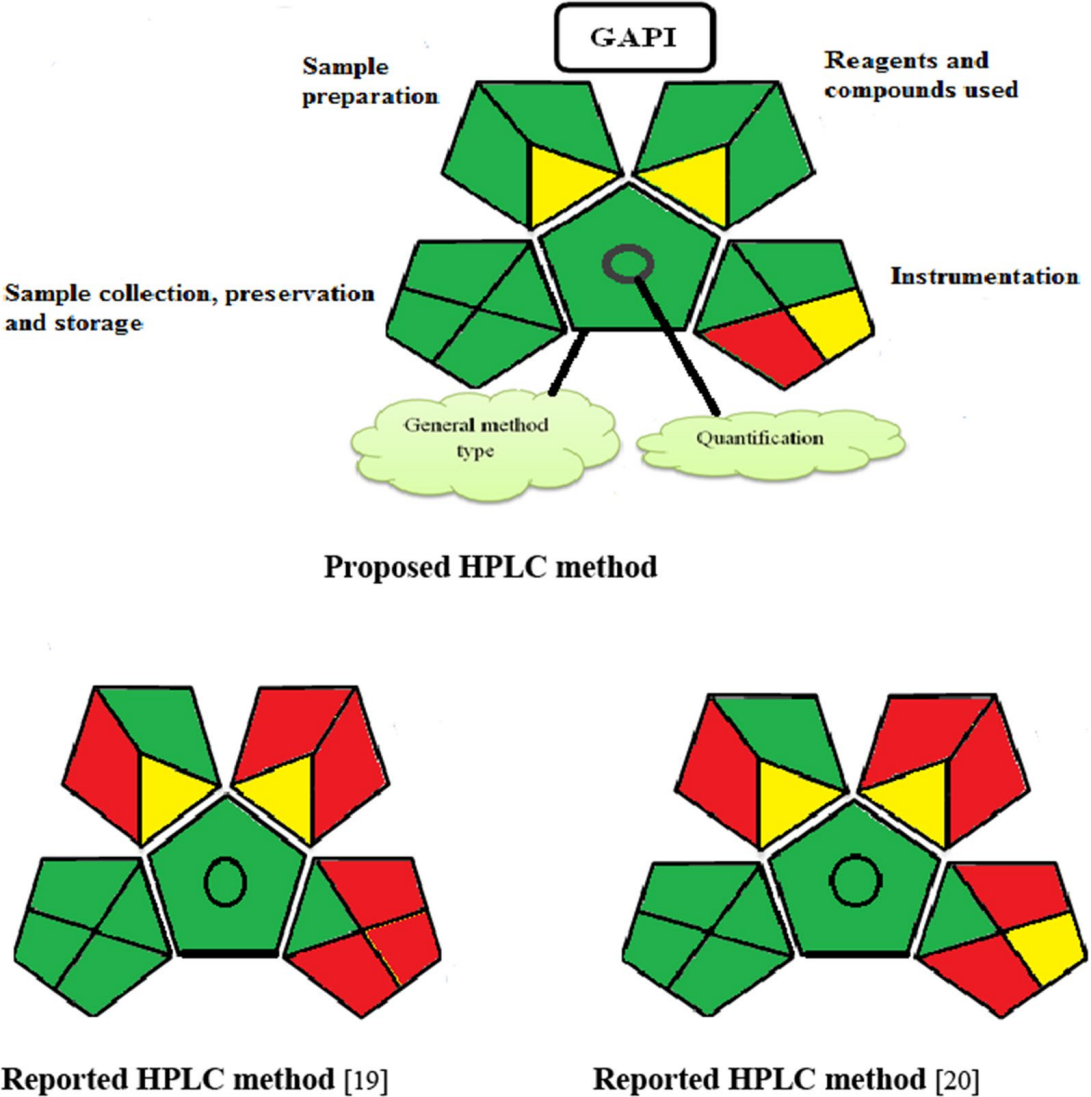
The green assessment report for the suggested HPLC method comparing to reported methods, using the GAPI tool

Second, Green Analytical Procedure Index (GAPI) [38] was also applied on the proposed and reported methods. The green assessment GAPI profiles for the proposed and reported HPLC methods are presented in Fig. 5.

Additionally, analytical Eco-scale was utilized for evaluating the proposed and reported methods, as represented in Table 10. The proposed method’s score was 95 which referred to an excellent green methodology (the closer the score to 100, the greener the method) [40].

**Table 10:** Eco-scale penalty points for the reported and the proposed HPLC methods [40]

Reported HPLC method [19]		Reported HPLC methods [20]		Suggested HPLC method	
Reagents	Penalty points	Reagents	Penalty points	Reagents	Penalty points
Methanol	18	Phosphate buffer	0	Ethanol	0
Formic acid	6	Potassium dihydrogen phosphate	3	Water	0
Water	0	Methanol	18		
	∑ 24		∑ 21		∑ 0

Instruments	Penalty points	Instruments	Penalty points	Instruments	Penalty points
HPLC–UV	1	HPLC–UV	1	HPLC–UV	1
Occupational hazard	0	Occupational hazard	0	Occupational hazard	0
Waste	12	Waste	9	Waste	4
	∑ 13		∑ 10		∑ 5
Total penalty points	37	Total penalty points	31	Total penalty points	5
Score	63	Score	69	Score	95

**Fig. 6:**
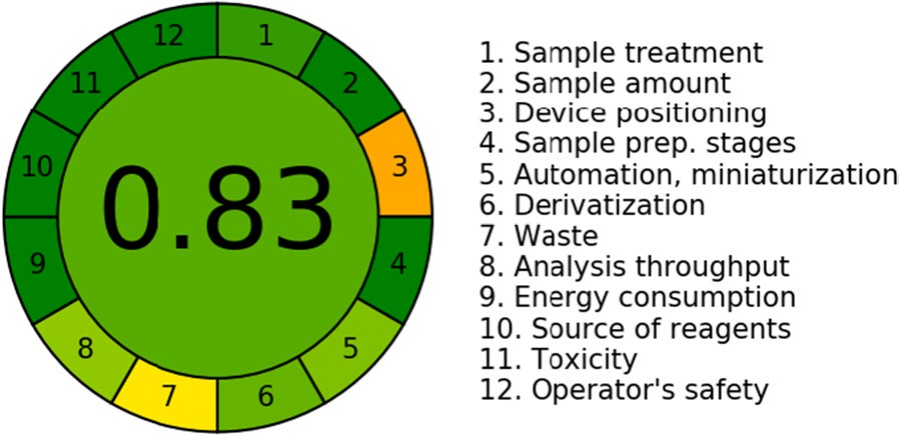
The evaluation of the proposed method greenness using analytical greenness metric (AGREE)

Finally, the greenness of the proposed method was investigated using AGREE-Analytical Greenness Metric Approach and software through evaluating 12 parameters of green analytical aspects. Figure 6 represents the twelve parameters with different colors ranging from dark green to orange based on information reported by Francisco Pena-Pereira et al. [41]. The score was found to be 0.83 indicating the greenness of the method (the closer the score to 1.0 the greener the method).

As described previously by the four assessment tools, it is concluded that the suggested HPLC technique has an environmental advantage over the two reported methods, and thus it could be employed for the routine analysis of PAR and DAN without affecting the environment.

## Conclusion

HPLC is the most commonly used technique for analysis of pharmaceutical compounds, so it is very important to minimize its bad effect as much as possible on both analysts and nature. However, most HPLC methods still do not consider the consequences of using unsafe compounds and solvents on the environment. The recommended mobile phase was mainly chosen for substituting unsafe solvents (such as methanol and acetonitrile) without influencing the chromatographic performance. The proposed technique for the determination of PAR and DAN was designed to avoid using harmful chemicals or create hazardous waste products in order to make it eligible for routine analysis. The proposed method was optimized and developed using a two-level FFD to predict the system suitability parameters. Employing FFD participated in decreasing the chemicals consumption, analysis steps and time. The recommended technique has low environmental impact which was ensured by investigating the method’s greenness using four assessment tools. Also, the proposed technique is rapid, repeatable and straight forward with no need for pretreatment. It was successfully applied for analysis of the studied drugs either in synthetic mixtures or combined capsules. All these benefits of the proposed method made it qualified to be used as a greener substitute for routine analysis of PAR and DAN in quality control laboratories and food analysis.

**Fig. 7:**
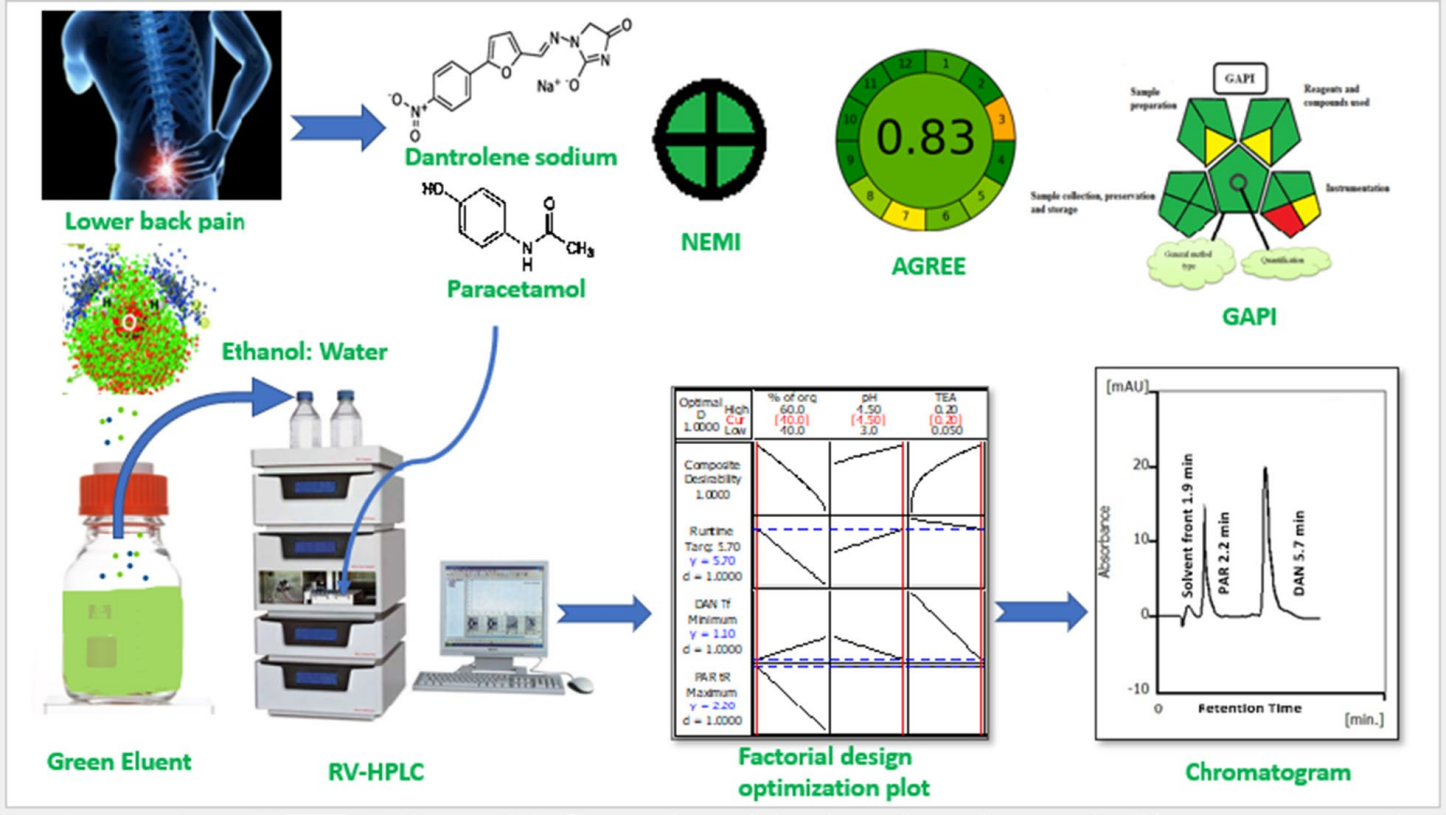
A graphical abstract that summarized the suggested approach

A graphical abstract that summarized the suggested approach was presented in Fig. 7.

## Supplementary Information

**Additional file 1:** **Table S1:** Suggested 8 runs to perform 23 experimental factorial designs. **Figure S1:** 2^3^ FFD pareto charts of the effects on the chromatographic responses at alpha = 0.05. **Figure S2, S3:** 2^3^ FFD main effect & full interaction plots for chromatographicresponses by data means type.

## Data Availability

All the data generated or analysed during this study are included in this article and its Additional file 1.
