# Supplementary material for: Hydro-organic mobile phase and factorial design application to attain green HPLC method for simultaneous assay of paracetamol and dantrolene sodium in combined capsules
Source: BMC Chem. 2023 Aug 2;17(1):92. doi: 10.1186/s13065-023-00990-7 (PMC10394946; doi:10.1186/s13065-023-00990-7)
Supplement: Supplementary file 1 — Additional file 1: Table S1: Suggested 8 runs to perform 23 experimental factorial designs. Figure S1: 23 FFD pareto charts of the effects on the chromatographic responses at alpha = 0.05. Figure S2, S3: 23 FFD main effect & full interaction plots for chromatographicresponses by data means type. [file 13065_2023_990_MOESM1_ESM.docx]

**Hydro-organic Mobile Phase and Factorial Design Application to Attain Green HPLC method for Simultaneous Assay of Paracetamol and Dantrolene Sodium in the Combined Capsules**

**Nora. A. Abdallah^*^, Manar M. Tolba, Amina M. El-Brashy, Fawzia A. Ibrahim, Mona E. Fathy**

Department of Pharmaceutical Analytical Chemistry, Faculty of Pharmacy,

Mansoura University, Mansoura 35516, Egypt.

**Table S1:** Suggested 8 runs to perform 23 experimental factorial designs.

**Figure S1:** 2^3^ FFD pareto charts of the effects on the chromatographic responses at alpha = 0.05.

**Figure S2, 3:** 2^3^ FFD main effect & full interaction plots for chromatographic responses by data means type.

**Table S1: Suggested 8 runs to perform 2^3^ experimental factorial designs.**

| **Run Number** | **% of organic modifier** | **pH** | **TEA** |
| --- | --- | --- | --- |
| 1 | 60 | 4.5 | 0.20 |
| 2 | 40 | 4.5 | 0.05 |
| 3 | 40 | 3.0 | 0.20 |
| 4 | 40 | 3.0 | 0.05 |
| 5 | 60 | 3.0 | 0.20 |
| 6 | 40 | 4.5 | 0.20 |
| 7 | 60 | 4.5 | 0.05 |
| 8 | 60 | 3.0 | 0.05 |


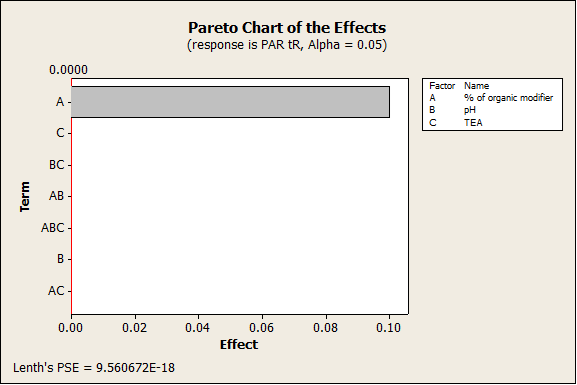

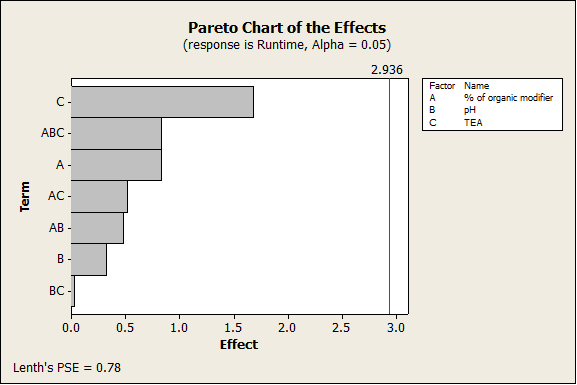

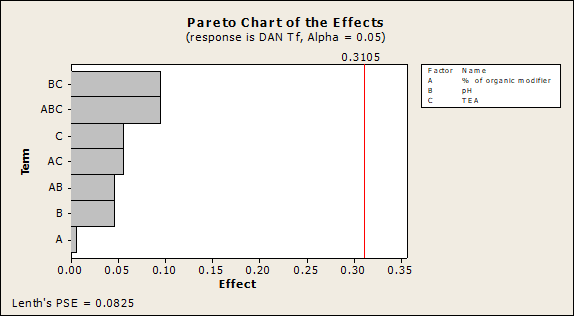
 **Figure S1**

**
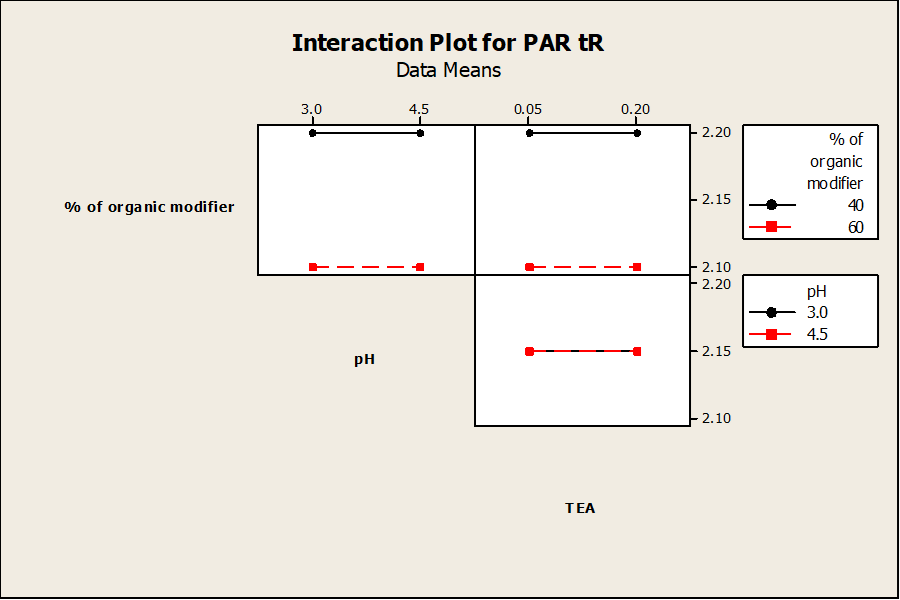
Figure S2**

**Figure S3**
